# Relevance of BCAR4 in tamoxifen resistance and tumour aggressiveness of human breast cancer

**DOI:** 10.1038/sj.bjc.6605884

**Published:** 2010-09-21

**Authors:** M F E Godinho, A M Sieuwerts, M P Look, D Meijer, J A Foekens, L C J Dorssers, T van Agthoven

**Affiliations:** 1 Department of Pathology, Josephine Nefkens Institute, Erasmus MC-University Medical Center Rotterdam, Rotterdam, 3000 CA, The Netherlands; 2 Department of Medical Oncology, Josephine Nefkens Institute, Erasmus MC-University Medical Center Rotterdam, Rotterdam, 3000 CA, The Netherlands; 3 Cancer Genomics Center, Josephine Nefkens Institute, Erasmus MC-University Medical Center Rotterdam, Rotterdam, 3000 CA, The Netherlands

**Keywords:** tamoxifen resistance, endocrine therapy, BCAR4, ERBB2, ERBB3, ERBB4

## Abstract

**Background:**

Breast cancer anti-oestrogen resistance 4 (*BCAR4*) was identified in a search for genes involved in anti-oestrogen resistance in breast cancer. We explored whether *BCAR4* is predictive for tamoxifen resistance and prognostic for tumour aggressiveness, and studied its function.

**Methods:**

*BCAR4* mRNA levels were measured in primary breast tumours, and evaluated for association with progression-free survival (PFS) and clinical benefit in patients with oestrogen receptor (ER*α*)-positive tumours receiving tamoxifen as first-line monotherapy for advanced disease. In a separate cohort of patients with lymph node-negative, ER*α*-positive cancer, and not receiving systemic adjuvant therapy, *BCAR4* levels were evaluated for association with distant metastasis-free survival (MFS). The function of BCAR4 was studied with immunoblotting and RNA interference in a cell model.

**Results:**

Multivariate analyses established high *BCAR4* mRNA levels as an independent predictive factor for poor PFS after start of tamoxifen therapy for recurrent disease. High *BCAR4* mRNA levels were associated with poor MFS and overall survival, reflecting tumour aggressiveness. In BCAR4-expressing cells, phosphorylation of v-erb-b2 erythroblastic leukaemia viral oncogene homolog (ERBB)2, ERBB3, and their downstream mediators extracellular signal-regulated kinase 1/2 and v-akt murine thymoma viral oncogene homolog (AKT) 1/2, was increased. Selective knockdown of ERBB2 or ERBB3 inhibited proliferation, confirming their role in BCAR4-induced tamoxifen resistance.

**Conclusion:**

BCAR4 may have clinical relevance for tumour aggressiveness and tamoxifen resistance. Our cell model suggests that BCAR4-positive breast tumours are driven by ERBB2/ERBB3 signalling. Patients with such tumours may benefit from ERBB-targeted therapy.

Over three decades, the anti-oestrogen tamoxifen has been the endocrine treatment of choice for patients with oestrogen receptor (ER*α*)-positive breast cancer (Early Breast Cancer Trialists' Collaborative Group, 1998). As an adjuvant therapy after surgery, tamoxifen reduces the incidence of relapse. In half of the patients with recurrent disease, tamoxifen induces an objective clinical response (Jaiyesimi *et al*, 1995; Jordan, 1995). However, the cancer will ultimately progress to hormone-independence that is, becoming unresponsive to tamoxifen. Despite extensive studies, the mechanisms involved in resistance are largely unknown (Dorssers *et al*, 1995; Clarke *et al*, 2003; Johnston *et al*, 2003; Nicholson *et al*, 2003; Osborne *et al*, 2005; Riggins *et al*, 2007).

Clinically, tamoxifen resistance is associated with poor prognosis and outcome. Thus, understanding of the mechanisms leading to this resistance is needed for developing new therapies. Previously, we applied several functional screens to identify genes involved in anti-oestrogen resistance (Dorssers *et al*, 1993; Van Agthoven *et al*, 1998; Brinkman *et al*, 2000; Meijer *et al*, 2006; Van Agthoven *et al*, 2009b). In one of the screens, we identified a new gene, breast cancer anti-oestrogen resistance 4 (*BCAR4*). In the tamoxifen-sensitive ZR-75-1 human breast cancer cell line, forced expression of *BCAR4* induced tamoxifen-resistant proliferation (Meijer *et al*, 2006).

To establish the clinical relevance of *BCAR4*, we investigated its relationship with tamoxifen resistance and cancer aggressiveness. In addition, we explored its biological function *in vitro*.

## Materials and methods

### RNA isolation, complementary DNA synthesis and quantification of mRNA transcripts

The isolation of RNA, quantification, complementary DNA synthesis, and normalisation to reference genes were performed as described before (Sieuwerts *et al*, 2005; details in the Supplementary Materials and Methods). TaqMan gene expression Assay-on-demand assays were *BCAR4* Hs00415922_m1, epidermal growth factor receptor (*EGFR*; Hs01076091_m1), v-erb-b2 erythroblastic leukaemia viral oncogene homolog *(ERBB)2* (Hs00170433_m1), *ERBB3* (Hs00176538_m1) and *ERBB4* (Hs00171783_m1) (Applied Biosystems International, Nieuwerkerk a/d Ijssel, the Netherlands), used according to the recommendations of the supplier.

### Cell lines

The ZR-75-1 and derived cell lines containing expression vectors for BCAR4 (ZR/BCAR4; Meijer *et al*, 2006), BCAR3 (ZR/BCAR3; Van Agthoven *et al*, 1998), EGFR (ZR/EGFR; Van Agthoven *et al*, 1992), v-akt murine thymoma viral oncogene homolog (AKT)1 (ZR/AKT1) and AKT2 (ZR/AKT2; Van Agthoven *et al*, 2009b), were cultured as described previously (Van Agthoven *et al*, 1992).

### Western blot analysis and immunoprecipitation

Immunoprecipitation and immunoblotting were performed as described previously (De Koning *et al*, 1996, details in the Supplementary Materials and Methods).

### Small interfering RNA-mediated inhibition of gene expression

Cells were seeded into 96-well plates at a density of 7500 per well. After 24 h, a mixture containing 25 *μ*l small interfering RNA (siRNA), 25 *μ*l DharmaFect3 (Perbio-Science, Etten Leur, the Netherlands), and 50 *μ*l medium was added. Final concentrations were 25 nM siRNA, 1 nM *β*-estradiol or 1 *μ*M 4-hydroxytamoxifen (Sigma-Aldrich Chemie, Zwijndrecht, the Netherlands). The ZR/EGFR cells were cultured in 4-hydroxyamoxifen-containing medium, with 10 ng ml^1^ EGF (Roche Diagnostics, Almere, the Netherlands). After 4 days, a WST-1 proliferation assay was performed (Roche Diagnostics). For each condition, six replicates were assayed. For RNA isolation, eight replicates were lysed with RNABee (Bio-Connect, Huissen, the Netherlands) and pooled. siRNAs were On TARGETplus SMARTpools (Dharmacon, Perbio-Science), each consisting of three oligonucleotides): EGFR (L-003114-00-0005), ERBB2 (L-003126-00-005), ERBB3 (L-003127-00-0005) and ERBB4 (L003128-00-0005).

### Clinical details

To assess the clinical relevance of *BCAR4* in breast cancer, we measured mRNA levels in a cohort of 1474 ER*α*-positive and -negative primary breast tumours from patients with detailed clinical follow-up (Van Agthoven *et al*, 2009a). BCAR4 was detected in 398 samples (27%). ER*α* status was determined by ligand-binding or enzyme immunoassays (Foekens *et al*, 1989), 73% of the tumours were ER*α*-positive. The medical ethics committee of the Erasmus MC-University Medical Center Rotterdam, the Netherlands, approved our study design (MEC 02.953). This retrospective study was in accordance with the Code of Conduct of the Federation of Medical Scientific Societies in the Netherlands, and is reported in line with the REMARK guidelines (McShane *et al*, 2006). All patients underwent surgery between 1979 and 1996.

To determine the association of *BCAR4* with tamoxifen resistance, samples from 280 patients (selected from the cohort of 1474 patients) with ER*α*-positive tumours, who received first-line tamoxifen therapy for advanced disease, were analysed. About 42% of these patients had lymph node-negative cancer and 10% presented with metastasis at diagnosis. None had received adjuvant hormone therapy. A total of 53 patients were treated with systemic adjuvant chemotherapy (21 with anthracycline- and 32 with non-anthracycline-based regimens). Response to tamoxifen treatment was monitored according to a standardised protocol (Hayward *et al*, 1977; EORTC Breast Cancer Cooperative Group, 2000). Clinical benefit, defined as objective (measurable) tumour response or no change for more than 6 months, was observed in 172 patients (62%) with 11 complete and 37 partial remissions, and 124 had no change for more than 6 months. From the remaining 108 patients, 91 had progressive disease and 17 had no change for 6 months or less. The median follow-up time after the start of tamoxifen therapy was 38.2 months. The median time that 50% of the patients experienced progression is 9.2 months.

For studying the relation between *BCAR4* mRNA levels and prognosis, 506 patients with lymph node-negative cancer, ER*α* protein-positive disease were selected from the cohort of 1474 patients. None received systemic adjuvant therapy. During follow-up, 193 experienced a relapse of distant metastasis (median follow-up time was 97 months). Patients with recurrent disease (115) were subsequently treated with tamoxifen. These were also included in the advanced study group of 280 patients.

### Statistical analyses

Statistical computations were performed with STATA, 10.1 (STATA Corp., College Station, TX, USA). Differences in mRNA concentrations were assessed by the Mann–Whitney *U* test or the Kruskal–Wallis test. Patient and tumour characteristics were used as grouping variables. Spearman rank correlation was used to quantify the strength of the monotonic association between continuous variables. For the levels of estrogen receptor (*ESR*)*1* and progesterone receptor (*PGR*), Box-Cox and logarithmic transformation was applied to reduce skewness. The transformed data were used for all analyses. The Cox proportional hazards model was used to calculate the hazard ratio (HR) and its 95% confidence interval in the analyses for metastasis-free survival (MFS), overall survival, progression-free survival (PFS) and post-relapse survival. For MFS, the end point was the first detection of a distant metastasis as confirmed after symptoms reported by the patient or at the time of detection of clinical signs at follow-up. This end point was preferred over relapse-free survival because relapse may be local and treated accordingly. The group is therefore more homogeneous from the perspective of treatment. During the years the tumours were collected (1979–1996), tumour grade was assessed by regional pathologists and not yet according current standards. In addition, approximately 30% of the pathology records lacked information on tumour grade. Therefore, we included in our prognostic analyses only the univariate survival data, because grade is included in the model for multivariate analysis. For all advanced patients treated with tamoxifen, PFS was defined as the time elapsed between initiation of first-line tamoxifen therapy and the first detection of disease progression. In multivariable analyses for PFS, the model included the classical predictive factors age, menopausal status at start of first-line therapy, the disease-free interval, the dominant site of relapse and *ESR1* and *PGR* mRNA levels. Proportional hazards assumption was verified by a test based on Schoenfeld residuals. In case of violation, the analysis was stratified for the variable. Data were visualised in survival curves with the method of Kaplan and Meier. The logrank test was used to compare survival curves, whereas for more than two groups the logrank test for trend was used. Logistic regression analysis was used for the relation between mRNA levels and clinical benefit of tamoxifen therapy and reported as the odds ratio and its 95% confidence interval. A two-sided *P*-value of <0.05 was considered statistically significant.

## Results

### Clinical relevance of BCAR4

#### Association of *BCAR4* mRNA levels with tamoxifen resistance

To address the question whether *BCAR4* is associated with clinical tamoxifen resistance, we studied 280 ER*α*-positive primary breast cancer specimens from patients with advanced disease. These patients received tamoxifen monotherapy as first-line treatment. The levels of *BCAR4* mRNAs were determined by quantitative RT–PCR of complementary DNA preparations of primary breast tumours. The levels of *BCAR4* mRNA were analysed for association with the clinicopathological factors and the end points PFS, clinical benefit and post-relapse survival. *BCAR4* mRNA was detected in 81 samples (29%). Tumours with mRNA levels below the detection limit were categorised as negative. Tumours with detectable levels of mRNA were categorised in a single group (positive), or in two groups (low or high) split at median levels. No relation between categorised *BCAR4* mRNA levels and age, menopausal status, tumour size, nodal status, or adjuvant systemic treatment of the patients was observed (Supplementary Table 1).

**Figure 1:**
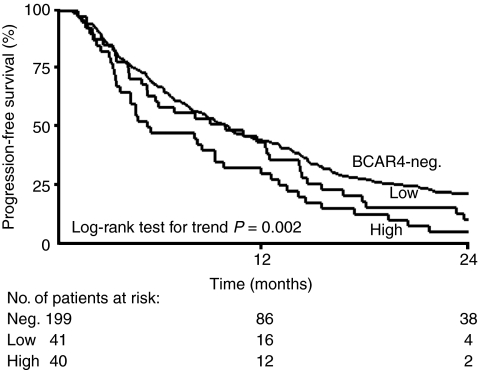
Progression-free survival of 280 patients with advanced disease treated with first-line tamoxifen monotherapy. Kaplan–Meier curves for PFS for subgroups of patients as a function of *BCAR4* mRNA status. Patients at risk at 12-month intervals are indicated.

Univariate Cox regression analysis revealed that the presence of *BCAR4* mRNA was significantly associated with shorter PFS (positive *vs* negative HR=1.45, *P*=0.007, Table 1). High levels were significantly associated with shorter PFS (high *vs* negative HR=1.70, *P*=0.003), whereas low levels were not informative. The Kaplan–Meier curves for PFS in these subgroups show rapid progression of the disease in patients with high levels of *BCAR4* (Figure 1).

In the multivariate analysis for PFS, high *BCAR4* levels were independently predictive for short PFS (high *vs* negative, HR=1.47, *P*=0.041, Table 1). In the univariate analysis of post-relapse survival, high *BCAR4* levels were related with poor outcome (high *vs* negative HR=1.68, *P*=0.007, Supplementary Table 2), but this association was not independent of the traditional predictive factors (HR=1.44, *P*=0.073). In the univariate logistic regression analysis of clinical benefit, high *BCAR4* levels were significantly associated with an unfavourable response to tamoxifen treatment (high *vs* negative odds ratio=0.49, *P*=0.042, Supplementary Table 3).

#### Association of BCAR4 mRNA levels with tumour aggressiveness

In our cell line model, expression of BCAR4 gives rise to an aggressive phenotype, that is, vigorous oestrogen-independent growth and anchorage independence (Meijer *et al*, 2006). Therefore, we investigated whether *BCAR4* mRNA levels give information on tumour aggressiveness. To estimate the true prognostic value of *BCAR4*, we performed analyses on a cohort of 506 patients with lymph node-negative cancer, ER*α*-positive disease. These patients had not received systemic adjuvant treatment. *BCAR4* mRNA was detected in 119 samples (24%). No relation between categorised *BCAR4* mRNA levels and age, menopausal status, tumour size, or tumour grade was found (Supplementary Table 4).

**Figure 2:**
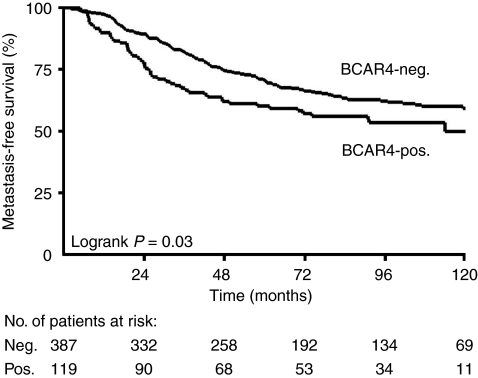
Metastasis-free survival in 506 patients with LNN, ER*α*-positive breast cancer. Kaplan–Meier curves for MFS for subgroups of patients as a function of *BCAR4* mRNA status of the primary tumours. Patients at risk at 24-month intervals are indicated.

Univariate analyses showed a significant association between the presence of *BCAR4* mRNA (positive *vs* negative) and shorter MFS (HR=1.41, *P*=0.033) and overall survival (HR=1.77, *P*=0.001; Table 2). Kaplan–Meier curves show the rapid recurrence of the disease in the group of patients with detectable *BCAR4* compared with the *BCAR4*-negative group (Figure 2). Patients with high BCAR4 in the tumours showed the worst MFS and overall survival (Table 2).

### Characterisation of BCAR4 using a breast cancer model

#### BCAR4 activates ERBB2 and ERBB3 signalling

**Figure 3:**
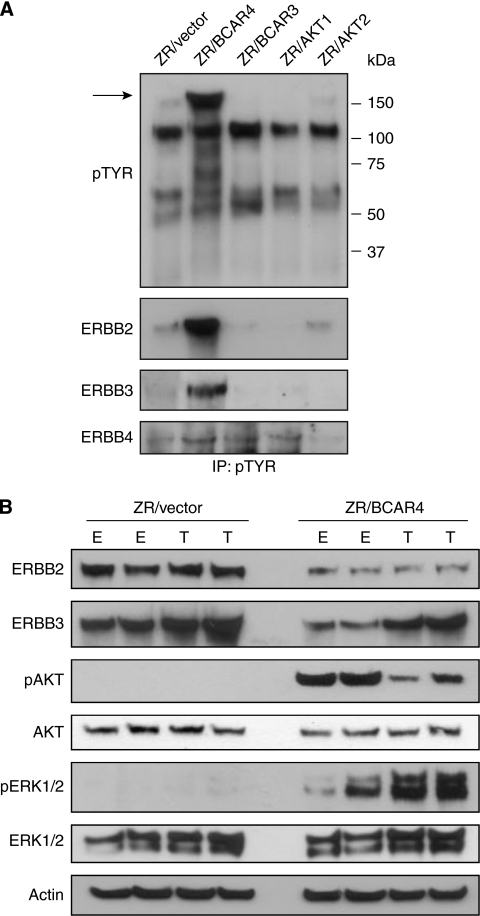
Activation of the ERBB2/ERBB3 signalling pathway by BCAR4. (**A**) Lysates of ZR-75-1 cells transduced with empty vector, *BCAR4*, *BCAR3*, *AKT1* or *AKT2* were immunoprecipitated with phosphotyrosine-specific antibody and subjected to western blot analysis. An approximately 180-kD band in lysates from ZR/BCAR4 cells is marked with an arrow. To identify the phosphorylated proteins in this band, identically loaded blots were probed with antibodies against EGFR, ERBB2, ERBB3 and ERBB4. Phospho-EGFR was not detected (data not shown). (**B**) Activation of downstream signalling of ERBB2/ERBB3. Lysates of two independent pools of ZR-75-1 cells containing empty vector or stably expressing *BCAR4*, cultured in the presence of oestradiol (E) or 4-OHT (T), were subjected to western blot analysis. Blots were probed with total ERBB2, ERBB3, phospho-AKT, total-AKT, phospho-extracellular signal-regulated kinase (ERK)1/2 and total-ERK1/2 antibodies and *β*-actin for loading control.

Identification of activated proteins in BCAR4-expressing cells may give important insight into BCAR4 function in anti-oestrogen-resistant proliferation. To identify proteins activated by BCAR4, we performed immunoprecipitation using an antibody directed to phosphorylated tyrosine residues. Lysates of ZR-75-1 cells were compared with lysates of equal numbers of cells with forced expression of BCAR4, BCAR3, AKT1 or AKT2. All these transgenic cell lines are resistant to tamoxifen, due to expression of the transgene (Van Agthoven *et al*, 1998; Brinkman *et al*, 2000; Meijer *et al*, 2006; Van Agthoven *et al*, 2009b). Among others, an abundant 180-kD band was detected in ZR/BCAR4 cells, which was hardly observed in the other cell lines (Figure 3A, arrow). The position of the protein band and the knowledge that the ERBB receptors can be involved in tamoxifen resistance provided a possible clue. Identically loaded blots were probed with antibodies against EGFR, ERBB2, ERBB3 and ERBB4. ZR-75-1 cells do not express EGFR (Van Agthoven *et al*, 1992) and phosphorylated EGFR was not detected. Low levels of phosphorylated ERBB4 were detected, but no increase in ZR/BCAR4 cells (Figure 3A). In contrast, phosphorylation of ERBB2 and ERBB3 was noticeably elevated in the ZR/BCAR4 cells (Figure 3A). This increase in phosphorylation of ERBB2 and ERBB3 in *BCAR4* transduced cells, compared with empty vector-expressing cells, was not due to higher total levels of these receptor proteins (Figure 3B). Additional analyses showed that AKT and extracellular signal-regulated kinase 1/2, representing major proliferative and survival pathways downstream of ERBB2/ERBB3 signalling, were also activated in ZR/BCAR4 cells (Figure 3B). This indicates that BCAR4 expression enhances the activity of the ERBB2 and ERBB3 receptors.

#### siRNA-mediated reduction of ERBB signalling inhibits BCAR4-induced proliferation

**Figure 4:**
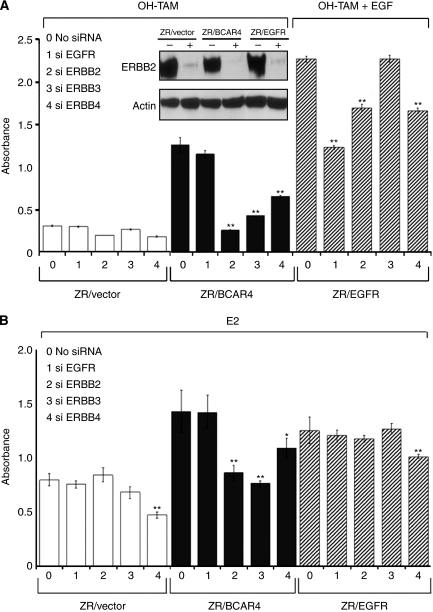
Knockdown of ERBB receptors reduces proliferation of cells with forced expression of *BCAR4* or *EGFR*. (**A**) Cells were exposed to 4-hydroxy tamoxifen (OH-TAM), plus EGF for ZR/EGFR cells. Insert shows the down-regulation of ERBB2 protein by western blot analysis, 96 h after treatment with siERBB2 (+) or transfection reagent only (−). (**B**) Proliferation was measured in oestradiol (E2)-containing medium. Bars represent the average of 6 independent siRNA transfections. Data of six replicates is reported as mean±s.d.. Significance was determined by the Mann-Whitney *U* test; ^*^P<0.05, ^**^P<0.01 compared to the control (medium without siRNA).

If increased ERBB2 and/or ERBB3 signalling is required for BCAR4-induced tamoxifen resistance then inhibition of ERBB2 and/or ERBB3 should suppress ZR/BCAR4 proliferation. To test this, ZR/BCAR4 and control cell lines were transfected with small interfering RNAs (siRNA) directed against the different ERBB receptors. Successful downregulation of ERBB2 was confirmed by western blot analysis (insert, Figure 4A). Inhibition of all ERBB receptors was verified by quantitative RT–PCR, showing reductions of over 50% of *EGFR* in ZR/EGFR cells, ⩾80% of *ERBB2* and *ERBB3*, and more than 60% for *ERBB4* mRNA transcripts.

The ZR-75-1 cells are oestrogen-dependent, and growth is inhibited by anti-oestrogens. As a consequence, addition of 4-hydroxytamoxifen inhibited proliferation of ZR-75-1 cells, irrespective of the addition of siRNA (Figure 4A). Knockdown of EGFR expression did not inhibit proliferation of ZR/BCAR4 cells, due to the fact that both our parental and ZR/BCAR4 cells are devoid of EGFR. However, *ERBB2*, *ERBB3*, and *ERBB4* siRNA all significantly inhibited proliferation of ZR/BCAR4 cells in the presence of 4-hydroxytamoxifen (Figure 4A). In the presence of EGF, ZR/EGFR cells are resistant to tamoxifen due to the expression of the *EGFR* transgene (Van Agthoven *et al*, 1992). Proliferation was reduced when EGFR was inhibited by siRNA-mediated interference (Figure 4A) confirming the involvement of this pathway in resistance of these cells. The knockdown of ERBB2 and ERBB4, but not ERBB3, in ZR/EGFR cells also resulted in the inhibition of proliferation.

In oestradiol-containing medium, proliferation of BCAR4-expressing cells was also inhibited by addition of *ERBB2* or *ERBB3* siRNA (Figure 4B). In contrast, knocking down ERBB2 or ERBB3 expression in the vector-control cells did not inhibit proliferation. In the presence of oestrogen, ZR/EGFR cells use the oestrogen receptor pathway and are not further stimulated with EGF. As expected, knockdown of EGFR in the presence of oestradiol did not inhibit proliferation (Figure 4B). In oestradiol-containing medium, proliferation of ZR-75-1, ZR/BCAR4 and ZR/EGFR cells was reduced by knockdown of *ERBB4*, suggesting a role of ERBB4 in oestrogen-regulated growth (Figure 4B).

## Discussion

We describe two sets of patients with ER*α*-positive breast cancer. The first group received tamoxifen as first-line treatment for advanced disease. This allowed for the analysis of clinical benefit, PFS and post-relapse survival in advanced breast cancer patients in relation to *BCAR4* mRNA levels in the primary tumour. In the second group, including only patients with lymph node-negative cancer, ER*α*-positive breast cancer, we investigated the prognostic value of *BCAR4* mRNA levels. Our data show that high *BCAR4* levels are independently predictive for shorter PFS after start of first-line tamoxifen therapy and in addition provide prognostic information for MFS and overall survival. Our results indicate that patients with high levels of *BCAR4* are at increased risk for early recurrence of the disease and have reduced probability of long-term benefit of tamoxifen treatment.

We show that ERBB2/ERBB3 signalling is critically involved in the mechanism of BCAR4-induced proliferation in the presence of oestrogen or tamoxifen. The overexpression of BCAR4 induced strong phosphorylation of ERBB2 and ERBB3. Key downstream mediators of ERBB signalling, AKT and extracellular signal-regulated kinase 1/2, were also activated in *BCAR4*-transduced cells. The siRNA-mediated inhibition of the ERBB receptors confirmed the direct involvement of ERBB2 and ERBB3 in BCAR4-mediated proliferation. The ERBB tyrosine kinase receptors have important roles in normal development, growth, and differentiation. Their involvement in numerous types of human tumours has been reported (for review, see Holbro *et al*, 2003). Gene amplification and overexpression of ERBB2 has been reported in several types of cancer, including the breast cancer, and has been shown to contribute to a poor clinical outcome (Slamon *et al*, 1989; Gullick *et al*, 1991; Seshadri *et al*, 1993; Berns *et al*, 1995; Liu *et al*, 2007; Van Agthoven *et al*, 2009a). Overexpression or amplification of ERBB2 predicts response failure to tamoxifen therapy (Wright *et al*, 1992; Berns *et al*, 1995; Dowsett *et al*, 2001; Hurtado *et al*, 2008; Van Agthoven *et al*, 2009a). In patients with hormone receptor-positive breast cancer who had received adjuvant tamoxifen therapy, EGFR, ERBB2 and ERBB3 phosphorylation was observed to be associated with shorter disease-free and overall survival (Frogne *et al*, 2009).

We have shown a mechanistic relationship between BCAR4 and the ERBB2/ERBB3 signalling pathways in the development of anti-oestrogen resistance *in vitro*. Therefore, BCAR4 expression may identify a subgroup of patients with increased ERBB2/ERBB3 signalling, independent of gene amplification and/or over expression of ERBB2. This is particularly important because currently the application of ERBB2-targeting drugs is limited to patients whose tumours express high levels of the ERBB2 protein or show amplification of the *ERBB2* gene (Hynes and Lane, 2005). Tumours with activated ERBB2 signalling via other mechanisms than amplification or over expression will be tested ERBB2-negative. These patients are currently withheld ERBB2-targeting drugs. However, it remains to be established whether *BCAR4*-positive primary tumours have elevated levels of phosphorylated ERBB2 or ERBB3.

The BCAR4 amino-acid sequence predicts two transmembrane domains, suggesting it may be localised at the cell membrane. Therefore, it might be a ligand for ERBB3, stimulating ERBB2/ERBB3 activity. It could also be a substrate for membrane-bound members of the ADAM (a disintegrin and metalloproteinase) domain family of proteins, some of them reported to be expressed in several cancers, including the breast cancer, in which they may release ERBB ligands and promote proliferation (for review, see Mochizuki and Okada, 2007). Another possible mechanism could be an intracellular interaction with the different ERBB receptors inducing their phosphorylation, as reported for nucleolin (Di Segni *et al*, 2008). The BCAR4 protein could also function similarly to Mucin4 that was reported to be a transmembrane ligand for ERBB2 (for review, see Carraway *et al*, 2001). Another ability of Mucin4 is to translocate ERBB2 to the apical surface in polarised epithelial cells (Ramsauer *et al*, 2003), or to increase the amount of ERBB2 and ERBB3 in the plasma membrane, by preventing their intracellular accumulation (Funes *et al*, 2006). How BCAR4 activates ERBB2/ERBB3 signalling remains to be established.

In conclusion, high levels of *BCAR4* mRNA predict resistance to endocrine therapy and poor outcome in ER*α*-positive breast cancer. If *BCAR4*-positive tumours are driven by ERBB2 signalling, in the absence of gene amplification or overexpression, as our results suggest, then more patients may benefit from ERBB2-directed therapy.

## Figures and Tables

**Table 1:** PFS after first-line tamoxifen treatment of 280 patients with oestrogen receptor-positive primary breast cancer

	**Univariate analysis**				**Multivariate analysis**		
	**No.**	**HR**	**95% CI**	***P***	**HR**	**95% CI**	***P***
*Age at start of therapy (years)*				0.083			0.109
⩽55	110	1.00			1.00		
56–70	102	0.85	0.64–1.12		0.74	0.49–1.10	
>70	68	0.70	0.51–0.96		0.62	0.40–0.96	
							
*Menopausal status at start of therapy*				0.454			0.216
Pre	73	1.00			1.00		
Post	207	0.90	0.68–1.19		1.30	0.86–1.96	
							
*Disease-free interval (years)* a				<0.001			
⩽1	72	1.00					
1–3	126	0.67	0.50–0.90				
>3	82	0.52	0.37–0.72				
							
*Dominant site of relapse*				0.673			0.517
Local regional relapse	29	1.00			1.00		
Bone	144	1.20	0.79–1.83		1.18	0.76–1.84	
Viscera	107	1.15	0.74–1.77		1.29	0.82–2.05	
*ESR1* mRNA level	280	0.91	0.86–0.96	0.001	0.92	0.86–0.98	0.010
*PGR* mRNA level	280	0.90	0.84–0.97	0.004	0.92	0.85–0.99	0.026
							
					**Additions to the base model^b^**		
*BCAR4*	280						
Positive *vs* negative	81/199	1.45	1.11–1.90	0.007	1.26	0.95–1.68	0.104
Low *vs* negative	41/199	1.27	0.90–1.80	0.177	1.12	0.78–1.60	0.554
High *vs* negative	40/199	1.70	1.20–2.41	0.003	1.47	1.02–2.13	0.041

Abbreviations: CI=confidence interval; HR=hazard ratio; PFS=progression-free survival.

*BCAR4* mRNA levels were defined as high, low or negative.

a Multivariate analyses were stratified for this variable.

b BCAR4 was introduced to the base model that included the factors age, menopausal status, dominant site of relapse and *ESR1* and *PGR* mRNA levels as transformed continuous variables. Inclusion of adjuvant chemotherapy in the base model did not change the estimates for *BCAR4.*

**Table 2:** Univariate analysis for metastasis-free and overall survival in 506 patients with oestrogen receptor-positive, lymph node-negative primary breast cancer

	**Metastasis-free survival**				**Overall survival**		
	**No.**	**HR**	**95% CI**	***P***	**HR**	**95% CI**	***P***
*Age (years)*				0.035			0.479
⩽40	56	1.00			1.00		
41–55	183	0.82	0.54–1.26		0.95	0.58–1.55	
56–70	159	0.61	0.39–0.96		0.91	0.55–1.51	
>70	108	0.53	0.32–0.89		1.26	0.74–0.215	
							
*Menopausal status*				0.054			0.334
Pre	203	1.00			1.00		
Post	303	0.76	0.57–1.01		1.16	0.86–1.58	
							
*Tumour size (cm)*				0.269			0.232
⩽2	233	1.00			1.00		
>2	273	1.17	0.88–1.56		1.20	0.89–1.62	
							
*Grade*				0.001			0.040
Poor	243	1.00			1.00		
Unknown	161	1.11	0.81–1.50		1.00	0.72–1.40	
Moderate	102	0.51	0.33–0.78		0.60	0.39–0.93	
*ESR1* mRNA	506	0.95	0.89–1.01	0.080	0.98	0.92–1.04	0.546
*PGR* mRNA	506	0.85	0.78–0.93	<0.001	0.84	0.76–0.92	<0.001
							
*BCAR4*							
Positive *vs* negative	119/387	1.41	1.03–1.94	0.033	1.77	1.28–2.45	0.001
Low *vs* negative	60/387	1.29	0.84–1.98	0.240	1.62	1.06–2.48	0.027
High *vs* negative	59/387	1.54	1.03–2.31	0.037	1.94	1.28–2.94	0.002

Abbreviations: CI=confidence interval; HR=hazard ratio; MFS=metastasis-free survival.

*BCAR4* mRNA levels were defined as high, low or negative.
