# Supplementary material for: Relevance of BCAR4 in tamoxifen resistance and tumour aggressiveness of human breast cancer
Source: Br J Cancer. 2010 Sep 21;103(8):1284–91. doi: 10.1038/sj.bjc.6605884 (PMC2967058; doi:10.1038/sj.bjc.6605884)
Supplement: Supplementary Materials and Methods [file 6605884x1.doc]

**Supplementary Table 1**

**Associations of *BCAR4* with Clinicopathological Factors in 280 Advanced Estrogen Receptor-Positive Patients**

***BCAR4***

**N Negative Low High *P***

**Age at surgery (y)** 0.72

≤ 55 129 90 22 17

55-70 102 76 12 14

>70 49 33 7 9

**Menopausal status at surgery** 0.65

Pre 99 69 17 13

Post 181 130 24 27

**Tumor size (cm)** 0.26

≤ 2 74 50 15 9

>2 206 149 26 31

**Nodal status** 0.30

0 118 84 17 17

1-3 67 50 13 4

>3 82 57 11 14

13 unknown

**Metastatic** 0.45

M0 251 177 39 35

M1 29 22 2 5

#### Grade 0.65

Poor 152 110 21 21

Good/moderate 35 23 8 4

Unknown 93 66 12 15

**Adjuvant therapy** 0.55

None 227 162 31 34

Chemotherapy 53 37 10 6

**PGR protein** 0.68

Negative 48 36 5 7

Positive 222 157 34 31

missing 10

**Supplementary Table 2**

## Post Relapse Survival after First-Line Tamoxifen Treatment of 280 Patients with Estrogen Receptor-Positive Primary Breast Cancer

## Univariate Analysis Multivariate Analysis

# N=280 HR 95% CI *P* HR 95% CI *P*

# Age at start of therapy (y) 0.958 0.902

≤ 55 110 1.00 1.00

56-70 102 1.04 0.77 to 1.41 0.96 0.61 to 1.52

> 70 68 1.04 0.73 to 1.48 1.05 0.64 to 1.71

### Menopausal status at start of therapy 0.612 0.631

pre 73 1.00 1.00

post 207 1.08 0.80 to 1.46 1.12 0.71 to 1.76

**Disease-free interval (y) †** <0.001

≤ 1 72 1.00

1-3 126 0.62 0.45 to 0.85

>3 82 0.45 0.31 to 0.67

### Dominant site of relapse 0.154 0.030

local regional relapse 29 1.00 1.00

bone 144 1.39 0.83 to 2.32 1.35 0.80 to 2.28

viscera 107 1.61 0.96 to 2.72 1.84 1.07 to 3.16

***ESR1*  mRNA** 280 0.92 0.87 to 0.98 0.014 0.91 0.85 to 0.98 0.014

***PGR* mRNA** 280 0.88 0.81 to 0.95 0.002 0.90 0.82 to 0.98 0.012

**Additions to the base model †**

***BCAR4*** 280

Positive vs. Negative 81/199 1.41 1.06 to 1.88 0.022 1.33 0.98 to 1.80 0.067

Low vs. Negative 41/199 1.22 0.85 to 1.77 0.285 1.24 0.84 to 1.82 0.284

High vs. Negative 40/199 1.68 1.15 to 2.46 0.007 1.44 0.97 to 2.14 0 .073

Abbreviations: HR, Hazard Ratio; CI, Confidence Interval. **BCAR4* mRNA levels were defined as High, Low or Negative. † Multivariate analyses were stratified for this variable. ‡ BCAR4 was introduced to the base model that included the factor age, menopausal status, dominant site of relapse and *ESR1* and *PGR* mRNA levels.

**Supplementary Table 3.**

**Clinical Benefit of First-Line Treatment with Tamoxifen of 280 Patients with Estrogen Receptor-Positive Primary Breast Cancer**

**Univariate Analysis Multivariate Analysis**

**No. OR 95% CI  *P* OR 95% CI *P***

**Age at start of therapy (y)**  0.255 0.997

≤55 110 1.00 1.00

56-70 102 1.54 0.88 to 2.68 1.04 0.44 to 2.41

>70 68 1.47 0.79 to 2.75 1.03 0.42 to 2.51

**Menopausal status at start of therapy**  0.104 0.545

pre 73 1.00 1.00

post 207 1.57 0.91 to 2.69 1.30 0.55 to 3.07

**Disease-free interval (y)** <0.001 0.950

≤ 1 72 1.00 1.00

1-3 126 3.26 1.78 to 5.95 0.92 0.53 to 1.60

>3 82 4.03 2.05 to 7.92 1.01 0.46 to 2.22

**Dominant site of relapse** 0.546 0.720

local regional relapse 29 1.00 1.00

bone 144 0.74 0.32 to 1.70 0.69 0.28 to 1.72

viscera 107 0.96 0.40 to 2.26 0.76 0.30 to 1.95

***ESR1* mRNA** 280 1.21 1.08 to 1.36 0.001 1.20 1.05 to 1.37 0.006

***PGR* mRNA** 280 1.14 0.99 to 1.31 0.079 1.07 0.91 to 1.26 0.405

**Additions to the base model ‡**

***BCAR4*** 280

Positive vs. Negative 81/199 0.61 0.36 to 1.04 0.068 0.78 0.44 to 1.37 0.386

Low vs. Negative 41/199 0.77 0.39 to 1.52 0.446 0.92 0.44 to 1.95 0.837

High vs. Negative 40/199 0.49 0.25 to 0.97 0.042 0.65 0.31 to 1.37 0.260

Abbreviations: OR, Odds Ratio; CI, Confidence Interval. *BCAR4* mRNA levels were defined as High, Low or Negative. ‡ BCAR4 was introduced to the base model that included the factor age, menopausal status, dominant site of relapse and *ESR1* and *PGR* mRNA levels.

**Supplementary Table 4.**

**Associations of *BCAR4* with Clinicopathological Factors in 506 Patients with Estrogen Receptor-Positive, Node-Negative Breast Cancer**

***BCAR4***

**N Negative Low High *P***

**Age at surgery (y)** 0.96

≤ 55 239 182 29 28

55-70 159 122 17 20

>70 108 83 14 11

**Menopausal status** 0.54

Pre 203 157 26 20

Post 303 230 34 39

#### Tumor size 0.72

≤ 2 cm 233 182 26 25

>2cm 273 205 34 34

**Nodal status** n.a.

0 506 387 60 59

1-3 0

>3 0

13 unknown

**Grade** 0.68

Poor 243 180 31 32

Good/moderate 102 80 13 9

Unknown 161 127 16 18

**Adjuvant therapy** n.a.

None 506 387 60 59

Chemotherapy 0

**PGR protein** 0.88

Negative 91 69 12 10

Positive 394 302 45 47

missing 21

n.a: not applicable

## Supplementary Materials and Methods

## RNA isolation, cDNA synthesis and quantification of BCAR4 mRNA transcripts.

Primary tumour tissues were processed as described previously (Sieuwerts et al. 2005). In summary, 20 to 60 cryostat sections (30 μm) corresponding to 30 to 100 mg of frozen tissue were cut and used for RNA isolation. To determine the amount of tumor cells relative to the amount of surrounding stromal cells, 5 μm sections were cut before, in between, and after cutting the sections for RNA isolation. These sections were then stained with haematoxylin and eosin. This study included only specimen with at least 30% epithelial tumour nuclei, uniformly distributed over at least 70% of the section area. Of both patient tissues and lysates from cell lines, RNA isolation, quantification of specific mRNA transcripts, cDNA synthesis and quality control checks were performed as described before (Sieuwerts et al. 2005). Real-time reverse-transcriptase PCR (RT-PCR) was performed on an ABI Prism 7700 Sequence Detection System, Nieuwerkerk a/d IJssel, the Netherlands). A TaqMan **BCAR4** gene expression Assay-on-demand Hs00415922_m1 (Applied Biosystems) was used according to the recommendations of the supplier. Primer sequences for *ESR1, PGR*, and the housekeeping genes, as well as how PCR reactions and validations were performed to ensure PCR specificity, have been described previously (Sieuwerts et al. 2005). The mRNA expression levels were relative to the expression levels of our set of reference genes, which included the low abundance hydroxymethylbilane synthase (*HMBS*), the medium abundance hypoxanthine-guanine phosphoribosyltransferase (*HPRT*) and the high abundance gene β-2-microglobulin (*B2M*). The mRNA expression levels were quantified as follows: mRNA target = 2^(mean Ct ref – mean Ct target)^, as described before (Sieuwerts et al. 2005).

**Western blot analysis and Immunoprecipitation**

Immunoprecipitation and immunoblotting were performed as described by De Koning et al.1996. Proteins were separated on 4-20% Precise Protein Gels (Pierce, Thermo Scientific, Etten-Leur, the Netherlands) and electro-blotted on Hybond PVDF membranes (Amersham Biosciences Benelux, Roosendaal, the Netherlands). Filters were blocked in PBS containing 0.1% Tween 20 and 5% Protifar (Nutricia, Zoetermeer, the Netherlands), incubated with primary antibodies overnight. Subsequently, these immunoblots were incubated with secondary horseradish peroxidase-conjugated antibodies (Amersham), which were detected using enhanced chemiluminescent substrate (Pierce). Antibodies used for immunoprecipitation and western blotting were anti-phosphotyrosine antibody 4G10; anti-EGFR; anti-ERBB3 and 4 (Upstate Biotechnology, Inc. Lake Placid NY); total Akt; p-Akt (Cell Signaling Technology, Inc. New England Biolabs, Hitchin UK); ERK1, 2; p-ERK1, 2 (Santa Cruz Biotechnology, Santa Cruz, CA); anti-ERBB2 (DakoCytomatation BV, Heverlee, Belgium); AKT2 and Beta-actin (Sigma).

References

1. Sieuwerts AM, Meijer-van Gelder ME, Timmermans M, Trapman AM, Garcia RR*, et al.* (2005) How ADAM-9 and ADAM-11 differentially from estrogen receptor predict response to tamoxifen treatment in patients with recurrent breast cancer: a retrospective study. *Clin Cancer Res* 11**,** 7311-7321.

2. De Koning JP, Schelen AM, Dong F, Van Buitenen C, Burgering BMT*, et al.* (1996) Specific involvement of tyrosine 764 of human granulocyte colony- stimulating factor receptor in signal transduction mediated by p145/Shc/GRB2 or p90/GRB2 complexes. *Blood* 87**,** 132-140.
